# Cardiac Cachexia - A Window to the Wasting Disorders

**DOI:** 10.5935/abc.20180009

**Published:** 2018-01

**Authors:** Andrew J Stewart Coats

**Affiliations:** IRCCS, San Raffaele-Pisana, Roma, Italy

**Keywords:** Cachexia, Wasting Syndrome, Exercise, Nutritional Physiological Phenomena

**To The Editor**

I read with interest the recent review by Okoshi and colleagues in the journal.^1^ This was a thoroughly enjoyable read that reviewed the main areas of focus. I would like, however, to reinforce some of the arguments. In the section on neurohormonal blockade there has also been a successful phase 2 trial of the fourth generation beta-blocker espindolol in cancer cachexia.^2,3^ Clearly beta-blockers can be helpful also in cardiac cachexia given their crucial role in heart failure in general. Other cardiovascular drugs are also being explored for their beneficial or protective effects on skeletal muscle. These include, as the authors point out, the ACE inhibitor Imidapril. Others including trimetazidine are also being studied.^4^ One issue of difficulty is that we are starting from the point of no effective therapies and testing therapies one by one. The true multi-system complexity of cachexia and yet its similarity across different organ failure syndromes implies it will be a multi-barrelled approach that may be needed to solve it. We may need to combine neurohormonal blockade, immune modulation, nutritional and exercise support with pro-anabolic agents to get real clinical benefits. Perhaps as the authors point out Cardiac Cachexia where several of these agents are already on board may be a good place to start. The time for a much greater focus on all cachexias, including of course cardiac cachexia, is truly here and now.^5^
