# Supplementary material for: Cardiac Cachexia - A Window to the Wasting Disorders
Source: Arq Bras Cardiol. 2018 Jan;110(1):102–3. doi: 10.5935/abc.20180009 (PMC5831311; doi:10.5935/abc.20180009)
Supplement: Related texts

# Reply

**Authors:** Marina P. Okoshi, Rafael V. Capalbo, Fernando G. Romeiro, Katashi Okoshi

We truly appreciate the comments on our review manuscript published in the journal.^1^ The authors reinforced our point of view by citing some papers published after the submission of our manuscript. We agree that we should immediately initiate a greater focus on cachexia of all causes aiming its prevention and treatment. While nutritional support has been long recommended for cachexia management, only more recently was exercise highlighted as a tool to manage muscle wasting and sarcopenia.^2-4^ As correctly pointed out, due the capacity to prevent body weight loss in heart failure patients with reduced ejection fraction, neurohormonal blockade has also been evaluated in non-cardiac cachexia. However, concerning other therapies such as immune modulation and pro-anabolic agents, there is no convincingly evidence for a positive response^3,5,6^ suggesting that additional studies are needed before we can effectively prevent and treat cachexia associated with different diseases including chronic heart and renal failure, cancer, and chronic obstructive pulmonary disease.

Marina P. Okoshi Rafael V. Capalbo Fernando G.
Romeiro Katashi Okoshi

## References

[1] Okoshi MP, Capalbo RV, Romeiro FG, Okoshi K (2017). Cardiac cachexia: perspectives for prevention and
treatment. Arq Bras Cardiol.

[2] Gomes MJ, Martinez PF, Pagan LU, Damatto RL, Cezar MD, Lima AR (2017). Skeletal muscle aging: influence of oxidative stress and
physical exercise. Oncotarget.

[3] Aversa Z, Costelli P, Muscaritoli M (2017). Cancer-induced muscle wasting: latest findings in prevention
and treatment. Ther Adv Med Oncol.

[4] Belloum Y, Rannou-Bekono F, Favier FB (2017). Cancer-induced cardiac cachexia: pathogenesis and impact of
physical activity (Review). Oncol Rep.

[5] von Haehling S, Ebner N, dos Santos MR, Springer J, Anker SD (2017). Muscle wasting and cachexia in heart failure: mechanisms and
therapies. Nat Rev Cardiol.

[6] Lima AR, Pagan LU, Damatto RL, Cezar MD, Bonomo C, Gomes MJ (2017). Effects of growth hormone on cardiac remodeling and soleus
muscle in rats with aortic stenosis-induced heart
failure. Oncotarget.
